# To rebound or not to rebound

**DOI:** 10.7554/eLife.31646

**Published:** 2017-10-06

**Authors:** Bethany A Stahl, Alex C Keene

**Affiliations:** Department of Biological SciencesFlorida Atlantic UniversityBoca RatonUnited States

**Keywords:** sleep, sex, behavior, *D. melanogaster*

## Abstract

Sexual arousal in flies counteracts the effects of sleep deprivation.

**Related research article** Beckwith EJ, Geissmann Q, French AS, Gilestro GF. 2017. Regulation of sleep homeostasis by sexual arousal. *eLife* **6**:e27445. doi: 10.7554/eLife.27445

How do we know we are tired and that it is time to sleep, and why can we go without sleep when we are excited? Usually, after a sleepless night, we make up for it the following day by taking a nap (if possible) or by going to bed earlier – a process referred to as rebound sleep. According to a long-standing model, this need to catch up on our sleep is modulated by two distinct mechanisms: the sleep homeostat, which controls how much we sleep, and the circadian clock, which dictates when we sleep (Borbély, 1982). While this model lays the foundation for understanding how sleep is regulated, it neglects a variety of other social, emotional and environmental factors that impact on sleep.

Sleep is highly conserved throughout the animal kingdom at both the genetic and the functional level. Some species are also known to skip sleep in favor of migration, mating or other social interactions. Flies, for example, can forgo sleep when they are exposed to mechanical stimulation or social interactions, which makes them a popular model for studying the regulation of sleep (Gilestro et al., 2009). Now, in eLife, Giorgio Gilestro of Imperial College and co-workers – Esteban Beckwith as first author, Quentin Geissmann and Alice French – report new insights into how sexual arousal in flies affects their need for sleep (Beckwith et al., 2017).

To examine how 'social sleep deprivation' affects rebound sleep, Beckwith et al. exposed the flies to different social scenarios. First, they placed a male fly into an arena that already contained a male resident. The presence of another male caused the resident to lose sleep, but he caught up via rebound sleep once the male intruder had been removed (Figure 1). The resident also lost sleep when a receptive female fly was introduced, but he did not catch up via rebound sleep once the female was removed. This suggests that the sexual arousal induced by the female fly was sufficient to override any need for the male to catch up after a sleepless night.

**Figure 1.**
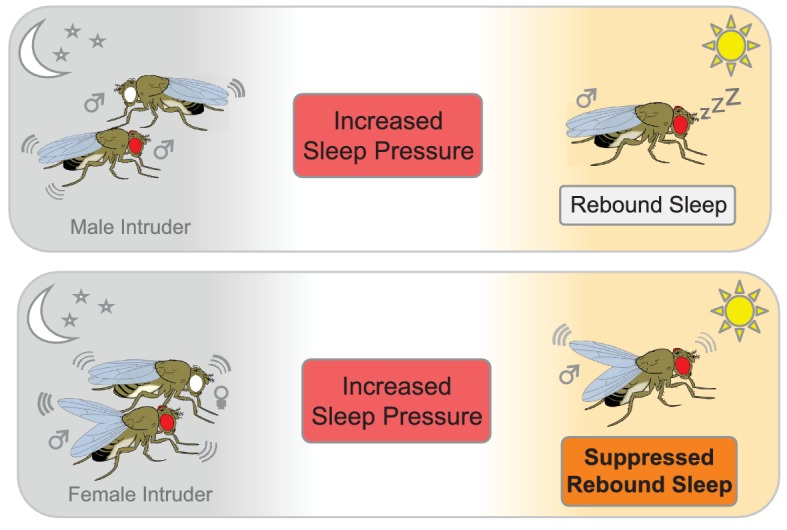
The effect of social interactions on rebound sleep in flies. Top panel: A male fly that has been deprived of sleep (red eye) by a male intruder (white eye) catches up on sleep the following day (rebound sleep). Bottom panel: Conversely, a male fly that has been deprived of sleep by pairing with a receptive female suppresses its need for sleep the following day, probably due to still being in a sexually aroused state (bottom panel).

What allows sexual arousal to overcome rebound sleep? Fly courtship is a multisensory experience that involves visual, tactile, acoustic and pheromonal cues. Beckwith et al. found that exposing male flies to female pheromones, or transferring them into tubes that previously contained a female fly, was sufficient to suppress rebound sleep.

To get to the bottom of why sexually aroused males did not catch up on lost sleep, Beckwith et al. looked deeper into the fly brain. Previous research has shown that male flies sense certain pheromones through neurons (and their receptor proteins) on their forelegs – this is why male flies repeatedly tap female flies with their legs during courtship. Beckwith et al. discovered that when males lacked the pheromone receptor *pickpocket 23* on these leg neurons, they did not notice the pheromones and rebound sleep occurred.

Moreover, the results showed that a specific cluster of neurons, called P1 neurons, are critical for courtship-suppressed sleep. When these neurons were stimulated, rebound sleep was inhibited. Taken together, these findings suggest that *pickpocket 23* neurons detect pheromones and then activate P1 neurons which, in turn, suppress sleep and prevent rebound sleep.

To better understand the mechanisms underlying the sexual arousal vs. sleep trade-off, we need to identify how pheromone circuits interface with sleep centers in the brain to modulate behavior. Two other recent papers shed light on this issue. In males, P1 neurons are activated by contact with females, and Chen et al. have shown that these neurons are connected with a set of wake-promoting neurons (Chen et al., 2017). In a separate study, Machado et al. discovered another pair of wake-promoting neurons that directly modulate courtship circuits (Machado et al., 2017).

Despite this progress, two central questions persist: how is the need for sleep sensed, and is sleep loss centrally integrated within the brain? So far, researchers have discovered many different neuronal circuits for sleep homeostasis within the fly brain, which could be directly or indirectly affected by sexual arousal and result in suppressed rebound sleep (Liu et al., 2016; Pimentel et al., 2016; Seidner et al., 2015). Identifying the neural circuits that regulate sleep and courtship will serve as a framework for determining the molecular sensors that know when we need to sleep.

Together, these findings highlight the integrated nature of sleep, and the way it is affected by the internal clock, the need for sleep and external factors, such as arousal. However, there is much that we do not know: for example, how do other external influences, such as stress, excitement or caffeine consumption, affect sleep loss and rebound sleep? Answering these questions will shed light on the basic functions of sleep.
